# Scaling‐down biopharmaceutical production processes via a single multi‐compartment bioreactor (SMCB)

**DOI:** 10.1002/elsc.202100161

**Published:** 2022-03-14

**Authors:** Lena Gaugler, Yannic Mast, Jürgen Fitschen, Sebastian Hofmann, Michael Schlüter, Ralf Takors

**Affiliations:** ^1^ Institute of Biochemical Engineering University of Stuttgart Stuttgart Germany; ^2^ Institute of Multiphase Flows Hamburg University of Technology Hamburg Germany

**Keywords:** cell culture, compartment discs, mixing times, multi‐compartment system, scale‐down

## Abstract

Biopharmaceutical production processes often use mammalian cells in bioreactors larger than 10,000 L, where gradients of shear stress, substrate, dissolved oxygen and carbon dioxide, and pH are likely to occur. As former tissue cells, producer cell lines such as Chinese hamster ovary (CHO) cells sensitively respond to these mixing heterogeneities, resulting in related scenarios being mimicked in scale‐down reactors. However, commonly applied multi‐compartment approaches comprising multiple reactors impose a biasing shear stress caused by pumping. The latter can be prevented using the single multi‐compartment bioreactor (SMCB) presented here. The exchange area provided by a disc mounted between the upper and lower compartments in a stirred bioreactor was found to be an essential design parameter. Mimicking the mixing power input at a large scale on a small scale allowed the installation of similar mixing times in the SMCB. The particularities of the disc geometry may also be considered, finally leading to a converged decision tree. The work flow identifies a sharply contoured operational field comprising disc designs and power input to install the same mixing times on a large scale in the SMCB without the additional shear stress caused by pumping. The design principle holds true for both nongassed and gassed systems.

**Abbreviations**
LC: lower compartment
SMCB: single multi‐compartment system

## 1 INTRODUCTION

Mixing plays an essential role in bioprocesses as it defines the environment that cells experience during cultivation [1]. The mixing process comprises of the homogenization of the liquid phase, dispersion of sparged gases to ensure sufficient mass transfer of oxygen and carbon dioxide, and transfer of heat [2]. However, during scale‐up, mixing times inevitably increase owing to biological and engineering constraints. This prohibits power inputs theoretically required to maintain short mixing times on a large scale [3].

With the reduction of the mixing quality on a large scale, there is an increase in the probability of the formation of heterogeneities [4]. Thus, as process development largely occurs in a well‐mixed lab scale, scale‐up does not accurately predict performance losses, and can even render a process economically unviable [5]. For biopharmaceutical processes, due to the high research and development costs, long time‐to‐market durations, and cost‐intensive production processes, these consequences might be even more pronounced [6].

To overcome the inconsistencies between development and production scale and thereby increase the accuracy of scale‐up, scale‐down systems have been used to study large‐scale effects at the lab scale. Large‐scale conditions are most often simulated in multi‐compartment systems consisting of several stirred tank reactors (STRs) or a combination of STR and plug‐flow reactors (PFR) connected by pump lines [7, 8]. For microbial processes, STR‐PFR systems are typically applied to study the effects of substrate and oxygen gradients on the production scale. More recently, similar systems have been adapted to simulate characteristic large‐scale effects in mammalian cell cultures, such as pH perturbations or hypoxia [4, 9, 10].

However, Nienow et al. showed that the inherent necessity of pumping cells through the system can deteriorate process performance when using a peristaltic pump, thereby masking the detrimental effects of large‐scale heterogeneities [11]. Furthermore, such systems are used to study the impacts of specific parameters rather than simulating large‐scale conditions more holistically. A different approach to create a multi‐compartment system at the lab scale was presented by Schilling et al. [12]. Artificial compartments were generated by installing cylindrical discs between the stirrers, prolonging the mixing times from 10 to 130 s. The authors outlined the application of this setup for investigating microorganisms exposed to large‐scale substrate gradients.

**PRACTICAL APPLICATION**

Due to the increase in research and development costs and decreasing approval rates in the biopharmaceutical industry, a deeper understanding of mammalian cells’ large‐scale behavior is essential. This study developed a single multi‐compartment bioreactor (SMCB) that allows the recreation of large‐scale mixing behavior on a small scale while avoiding increased contamination risks and shear stress caused by pumping. Thus, the SMCB could serve as a tool to unravel the metabolic mechanisms induced by the cultivation environment at the manufacturing scale. Further applications in cell line development and early stage bioprocess development are conceivable by integrating this tool in cell line selection to enable robust scale‐up.

The current study builds on the previous findings of Schilling et al. by elucidating whether and how such settings may be applicable for the scale‐up analysis of mammalian cells. A single multi‐compartment bioreactor (SMCB) was designed to enable tailoring of mixing times, similar to large‐scale conditions of biopharmaceutical production processes. Intrinsically, the approach differs from conventional multi‐compartment settings as it prevents any additional stress impacts caused by external pumps. Conceptually, large‐scale mixing times and (integral) volumetric power inputs guide the design of the laboratory scale‐up simulator. Particular emphasis was placed on the design of proper separating discs mounted inside the stirred bioreactor. Thorough studies will converge in a design workflow that allows proper downscaling of large‐scale mixing conditions into a 3.8 L lab bioreactor.

## 2 MATERIALS AND METHODS

### 2.1 Reactor setup and disc construction

**FIGURE 1:**
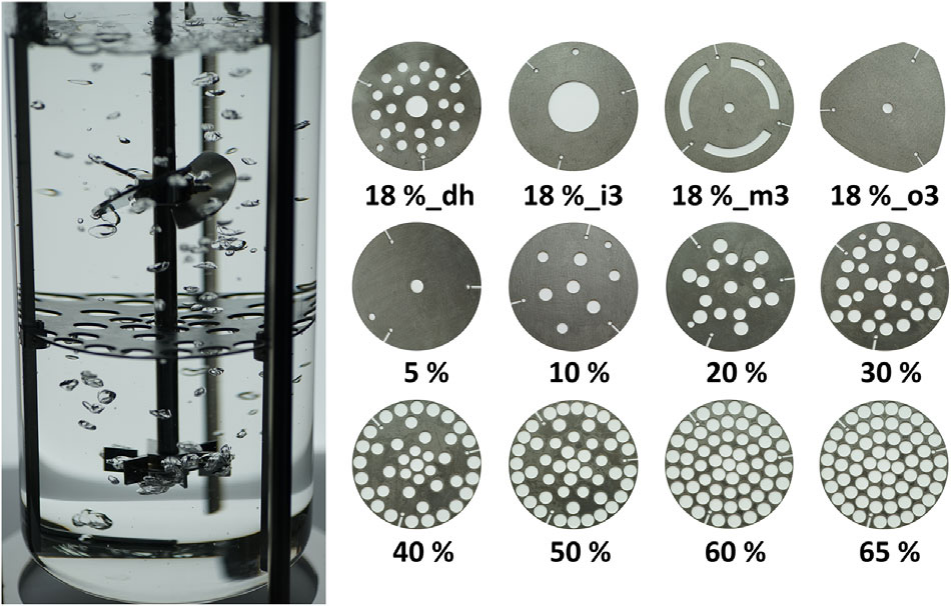
Single multi‐compartment bioreactor (SMCB) and (V4A stainless steel) compartment discs. The installed disc divides the liquid column into similar‐sized compartments (V_fill _= 3.23 L, H/D = 2). A combination of a Rushton turbine (bottom) and pitched‐blade turbine (top) was used as the impeller configuration. Discs differ in the exchange area provided between the compartments (values in %) or in the disc layout considering distributed holes (dh) and the inner (i3), middle (m3), and outer (o3) thirds of the radius

The compartment installations were integrated into a 3.8 L stirred tank glass reactor (Eppendorf, Hamburg, Germany). The reactor was equipped with a pitched‐blade impeller (top) and a Rushton turbine (bottom) to use a typical impeller configuration for mammalian cell culture applications. To enable easy retrofitting of bioreactors, compartment installations were designed as a self‐supporting system that requires no further screw connections to the reactor. The compartment discs rested on fixation blocks that can be screwed to the baffles at any desired height. Therefore, the baffles and fixation blocks served as universally applicable mounts for the discs. In this study, a single disc was installed in the middle of the liquid column to divide the reactor content into two similar‐sized compartments. The compartment discs, baffles, and fixation blocks were designed using AutoCAD 2021 (Autodesk Inc., CA, USA) and manufactured from V4A stainless steel via water jet cutting (SNZ Schneidebetrieb GmbH, Mühlacker, Germany). The designed discs (Figure 1) differed either in the exchange areas provided between the compartments (5–65% of the total cross‐sectional area) or in the disc layout. To elucidate the layout impacts, the same exchange area of 18% was distributed differently over the disc. Designs considered the inner (i3), middle (m3), and outer (o3) third of the radius, as well as distributed holes (dh).

### 2.2 Mixing time determinations

The mixing times were determined using an optical method based on the pH‐induced color change of bromothymol blue [13]. The reactor was filled with 3.23 L (aspect ratio: H/D = 2.0) of a 0.9% NaCl solution and 5 mL of a 1% (w/w in water) bromothymol blue solution. NaOH (2 M) and HCl (2 M) were used as the base and acid, respectively (Carl Roth, Karlsruhe, Germany). To prevent the formation of a carbonate buffer system, the reactor content as well as the acid and base were gassed with N_2_ to remove dissolved CO_2_. The reactor was then sealed to prevent the subsequent entry of CO_2_. In accordance with Godleski and Smith [14], the macromixing time was determined at an acid‐to‐base ratio of 5. The color change was recorded with a camera (Sony α7) at a frame rate of 25 frames/s. For the subsequent automated evaluation of the mixing time, the gray value progression was averaged per compartment. Mixing times were defined as the time needed to achieve 95% of the final gray value starting from the time point of acid addition.

### 2.3 M‐Star CFD simulations

The transient fluid motion was studied using the commercial software M‐Star CFD 2.10.62 (https://mstarcfd.com/). The underlying lattice Boltzmann method (LBM) uses a simplification of the Boltzmann transport equation to solve the Navier‐Stokes equations. The fluid was assumed to consist of interacting molecular parcels. Given a sufficiently large quantity, they can represent flow behavior. The number of molecules at each node is correlated to the local fluid density and changes owing to the collision and streaming steps.

(1) $$\begin{array}{c} \frac{f_{i}\left(x+c_{i}\Delta t,t+\Delta t\right)-f_{i}\left(x,t\right)}{\Delta t}=-\frac{1}{\tau }\left[f_{i}\left(x,t\right)-f_{i}^{eq}\left(x,t\right)\right] \end{array}$$

The standard lattice Boltzmann equation [15, 16] with a single relaxation time τ consists of the distribution function $f_{i}\left(x,t\right)$ associated with the *i* ^th^ velocity direction of compound *c*. During the lattice time step (Δt), the distribution moves towards the local equilibrium distribution function $f_{i}^{eq}\left(x,t\right)$.

A square lattice velocity model is composed of a stable velocity vector set that distributes the nodes equidistantly over the geometry. In the 3‐dimensional space, each node can reach 19 of its direct neighboring nodes per time step, which corresponds to a D3Q19 lattice field. A Δt of 3.12e^−5^ s maintained a lattice density deviation below 1.5%. The chosen lattice spacing of Δx of 5.20e^−4^ m resulted in a total of 31.2 million node points. A large‐eddy simulation (LES) was employed to model turbulence. Eddies smaller than the size of the lattice were calculated with the sub‐grid model suggested by Smagorinsky [17], using the default M‐Star value of 0.1 for the Smagorinsky coefficient. The interaction between the stirrer and the fluid was implemented using the immersed boundary method [18]. The reactor wall was considered to be a no‐slip condition. Each stirrer speed was simulated for 10 s, and the fluid properties on five output planes were recorded every 0.1 s.

### 2.4 Vertical velocity analysis

One output plane was implemented in accordance with the position of the compartment disc in the SMCB. The recorded vertical fluid velocities were exported as a 250 × 250 matrix for each simulated time step and averaged per stirrer speed.

The discs were converted to 250 × 250 binary matrices, with 0 representing solid disc material and 1 indicating positions that are available for fluid exchange. Using elementwise multiplication, new matrices were generated that only contained velocity values for the positions of fluid exchange on the respective disc. Therefore, the mean net velocities (${\bar{v}}_{i}$) were calculated neglecting all zeroes. In essence, the ${\bar{v}}_{i}$ values represent the resulting net velocity after integrating all feasible exchange flows for a given disc design.

### 2.5 P/V and mixing times in large scale

Two important nondimensional values for understanding, describing, and scaling a process to smaller or larger scales are: (i) the stirrer Reynolds number, which states the flow regime inside an agitated tank, and (ii) the power number, which reveals the agitator power input (*P*) into a system. Their relationship characterizes the power input of a specific impeller type. Although there are multiple scale‐up strategies, keeping the volume‐specific power input P/V constant is considered a good compromise for biopharmaceutical processes [19]. Eventually, there are two ways to obtain the power draw of the system. On one hand, the power number (*Po*) can be estimated according to published values and substituted in *P = Po ρ n^3^ d^5^*, where *ρ* is the fluid density, *n* is the stirrer frequency, and *d* is the impeller diameter. On the other hand, and more precisely, the stirrer torque (*M_stirrer_*) can be identified directly by a torque measuring cell (Lorenz DR‐3000) at the stirrer shaft. The latter leads to the total power input as

(2) $$P=2\pi M_{stirrer}n$$

and has successfully been demonstrated in previous studies [20, 21, 22]. The approach was used to measure and characterize the power input in an industrial‐scale acrylic glass reactor with a working volume of 15 m^3^. It was equipped with a bottom‐mounted magnetic agitator (ZETA BMRF), which allows the use of multiple stirrer combinations and numbers to mimic industrial cultivation.

Another scrutinized process criterion is the mixing time, which is visualized in the reactor using the decolorization method with phenolphthalein as presented by Rosseburg et al. [21]. For a pH lower than 8.2, the solution was colorless, whereas it turned pink for higher values. The addition of NaOH or HCl shifted the pH, which was recorded with a camera (Nikon D7500) and a lens (Carl Zeiss ZF Planar 1.4 50 mm) at a frame rate of 60 Hz. Spotlights and a white diffusor foil provided homogeneous background illumination. Finally, the gray‐scale values over time were calculated from the RGB images using an in‐house MATLAB image‐processing code [13, 22].

## 3 RESULTS AND DISCUSSION

The goal of this study was to identify an easy‐to‐implement procedure that enables the scaling down of an industrial process into a SMCB. In a previous study, mixing times were evaluated for a working volume of 12,000 L (H/D = 2, Rushton Turbine, Pitched‐blade turbine) and were found to be between 100 and 170 s for P/V values of 170 and 10 W/m^3^, respectively [22]. These physical values were used for the multi‐compartment design of the scale‐down bioreactor, which may serve as a working example for related applications.

### 3.1 Mixing time results with different compartment discs

First, fundamental studies were conducted to identify the aspects of disc design that allow the manipulation of mixing qualities. Mixing times were determined for a series of different compartment discs in a nongassed system. The discs varied in the exchange area (*A_ex_*) provided between the compartments and in the location where this exchange was installed on the disc (Figure 1).

**FIGURE 2:**
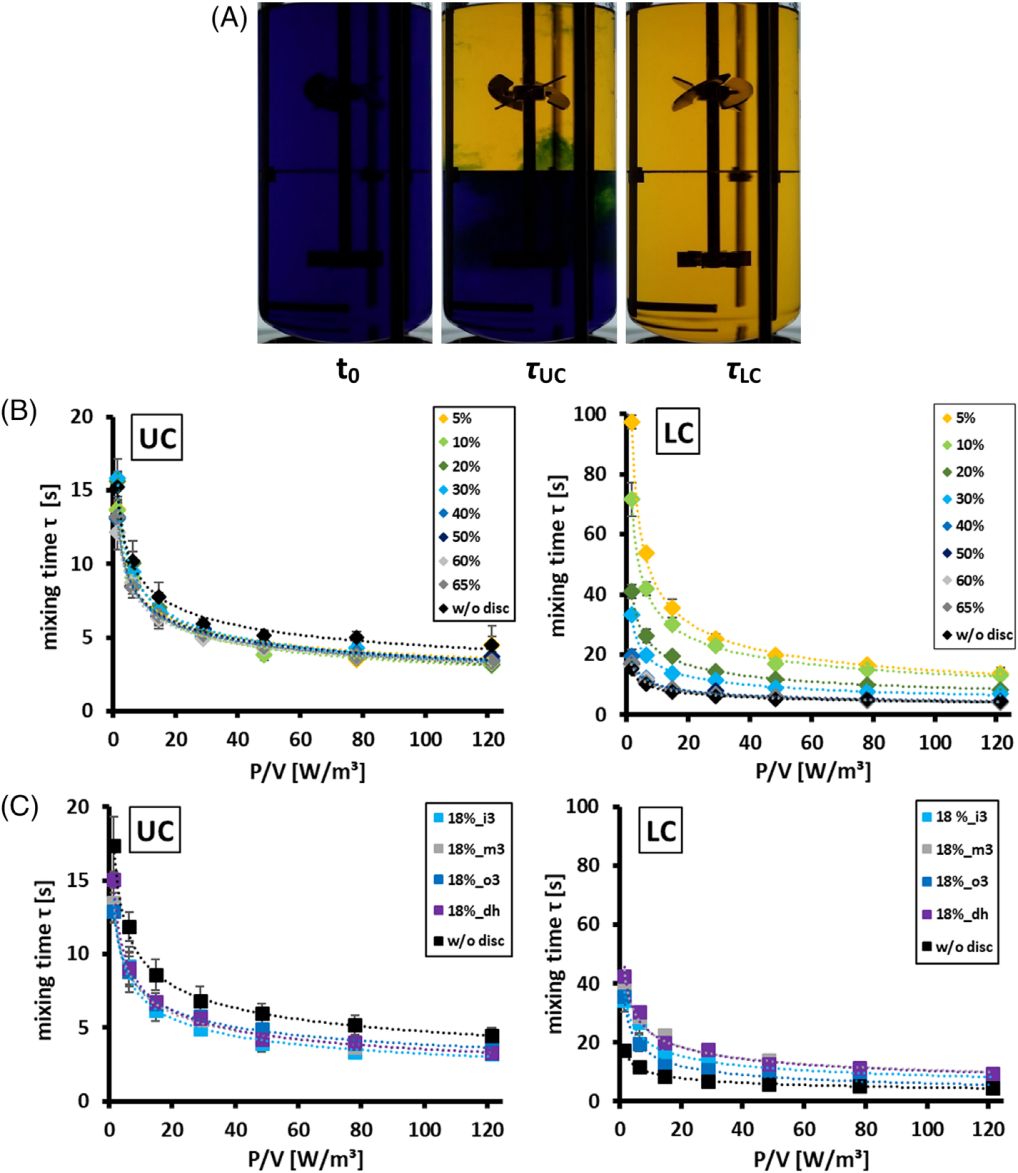
(A) Example of video frames recorded during mixing time experiments for the starting point (t_0_), the mixing time of the upper compartment (τ_UC_), and the mixing time of the lower compartment (τ_LC_). (B) Mixing time results in a non‐gassed system for the upper (UC) and lower compartments (LC) with compartment discs providing 5–65% of the total cross‐sectional area as exchange area as well as mixing times in the reference setup without a disc. (C) Nongassed mixing time results for the UC and LC with discs providing an exchange area of 18% in the inner (i3), middle (m3), or outer (o3) third of the radius or as distributed holes (dh). Error bars indicate the standard deviation (*n* = 5)

Figure 2A shows the mixing times for the upper and lower compartments (LC) with a characteristic compartment disc. As the mixing process of the two compartments overlapped, the starting point of mixing in the LC could not be specified. Therefore, as the last point of mixing was always located in the LC, the mixing times given for the LC corresponded to the mixing times of the entire reactor. The mixing times in the upper compartment were comparable for all discs tested. Hence, the LC mixing times determined the total mixing time in the two‐compartment setup.

An experimental series was conducted focusing on the impact of *A_ex_* provided between the compartments (Figure 2B). As indicated, the exchange areas ranged from 5 to 65%. Additionally, different mixing power inputs were investigated, covering settings of 1.5 to 121 W/m^3^. The range was chosen as it reflects the typical operational modes in biopharmaceutical processes with mammalian cells [23].

A clear correlation between *A_ex_* and the mixing times was observed. The more the exchange between the compartments was limited, the longer was the mixing time. Thus, the mixing times prolonged from 15.3 s at 1.5 W/m^3^ to up to 97.3 s. Other studies have reported large‐scale mixing times of 200–360 s in mammalian cell cultures [10, 24]. However, it must be kept in mind that the mixing times discussed in this study are macro‐mixing times. Time‐scales for achieving homogeneity at the molecular level can be expected to be considerably longer [25].

In addition, the mixing time evaluations in this study were limited to two similar‐sized compartments, as described previously for a 15,000 L bioreactor [21]. However, because of the intrinsic flexibility of the multi‐compartment concept applied here, more complex systems with higher numbers or different sizes of compartments can be realized. Thus, if required, additional discs could be inserted to push the mixing times to further extremes and generate, for example, additional dead zones or highly persistent feeding zones.

To achieve a noticeable difference in the obtained mixing times, the fluid exchange had to be limited to below a certain threshold. For the discs tested, an *A_ex_* ≤ 30% was required to obtain statistically significant changes (*p* = 0.05) in the mixing times compared to those in the reactor without a disc. With increasing power inputs, the mixing times approached constant values, and the differences between the discs diminished.

To evaluate the impact of different exchange locations on the disc, disc designs were varied at a constant *A_ex_*. The latter remained at 18%, whereas the disc designs differed according to the layout, as depicted in Figure 1. Again, the same range of mixing power input was screened to compare the results with those of the previous series. The examined modifications in the disc layout led to smaller but significant changes in the mixing times. The shortest mixing times were generated with disc 18%_o3, which limited the fluid exchange to areas near the vessel wall. The highest increase in the mixing times was created with the discs 18%_dh and 18%_m3, which reduced the vertical mixing near the vessel wall and in the proximity of the stirrer shaft, respectively.

### 3.2 Empirical correlations

As a first step to establish a correlation between *A_ex_* and the obtained mixing times, empirical functions between the mixing time (τ) and the volumetric power input were determined for each disc via linear regression. Equation (3a) represents a commonly applied approach for modeling the mixing time in a stirred vessel mixing a nonviscous medium [26].

(3a) $$\tau =k\;\cdot {\left(\frac{P}{V}\right)}^{\alpha }$$

The logarithmic version Equation (3b) allows for an easy identification of the regression parameters *k* and α, which was performed by least‐squares regression (Supporting information Table S1).

(3b) $$\ln\tau =\alpha \;\cdot \ln\left(\frac{P}{V}\right)+\ln k$$

**FIGURE 3:**
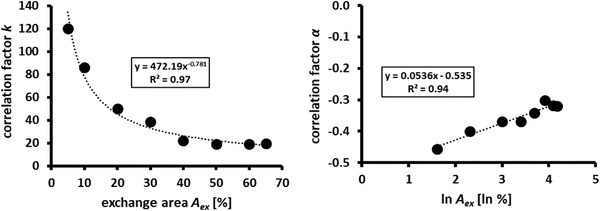
Factors *k* and *α* derived from empirical correlations between mixing time τ and power input (P/V) for different compartment discs versus exchange area (*A_ex_*), provided by the respective compartment discs

However, *k* and α should be correlated with the design parameter *A_ex_* while designing a scaled‐down multi‐compartment bioreactor. Consequently, *k* and α listed in Table S1 were plotted against *A_ex_* (Figure 3). With R^2^ values of 0.97 and 0.94, a statistically sound exponential correlation between *k* and *A_ex_* (Equation (4a)) and a linear correlation between α and *A_ex_* (Equation (4b)) were observed. The identified correlations are as follows:

(4a) $$k=\delta \cdot {A_{ex}}^{\varepsilon }$$

(4b) $$\alpha =\beta \cdot \ln A_{ex}+\gamma$$

By replacing α and *k* in Equation (3b) with the formulae (4a and 4b), Equation (3b) can be extended to implement the dependency from *A_ex_*.

(5) $$\ln\tau =\left(\beta \cdot \ln A_{ex}+\gamma \right)\;\cdot \ln\left(\frac{P}{V}\right)+\ln\left(\delta \cdot {A_{ex}}^{\varepsilon }\right)$$

Equation (5) can be repositioned for *A_ex_*. Now, τ should be taken as a constraint from large‐scale measurements, as this study aimed at mimicking large‐scale conditions in a small‐scale multi‐compartment bioreactor, that is, *τ = τ_large_*. By analogy, the scale‐up criterion of equal power input is often applied in mammalian cell culture bioprocesses. It is noteworthy that the related mixing power inputs are much lower than those of microbial bioprocesses, which renders this criterion applicable to cell culture processes. Hence, one may call for equal mixing power input on a small scale, that is P/V* = *P/V_large_.

By solving this correlation for *A_ex_*, the resulting equation allows the calculation of the *A_ex_* required to realize the desired mixing times (*τ*_large_) at volumetric power inputs used in large scale ((P/V)_large_) applications.

(6) $$A_{ex}={\left(\left(\tau_{large}\cdot \frac{1}{{\left(\frac{P}{V}\right)}_{large}^{\gamma }}\right)\frac{1}{\delta }\right)}^{\frac{1}{\beta \cdot \ln{\left(\frac{P}{V}\right)}_{large}+\varepsilon }}$$

with *A_ex_* is in [%], *τ*_large_ is in [s], (P/V)_large_ is in [W/m^3^], and *β, γ, δ*, and *ε* represent system‐specific correlation factors.

### 3.3 Evaluation of vertical fluid velocities

**FIGURE 4:**
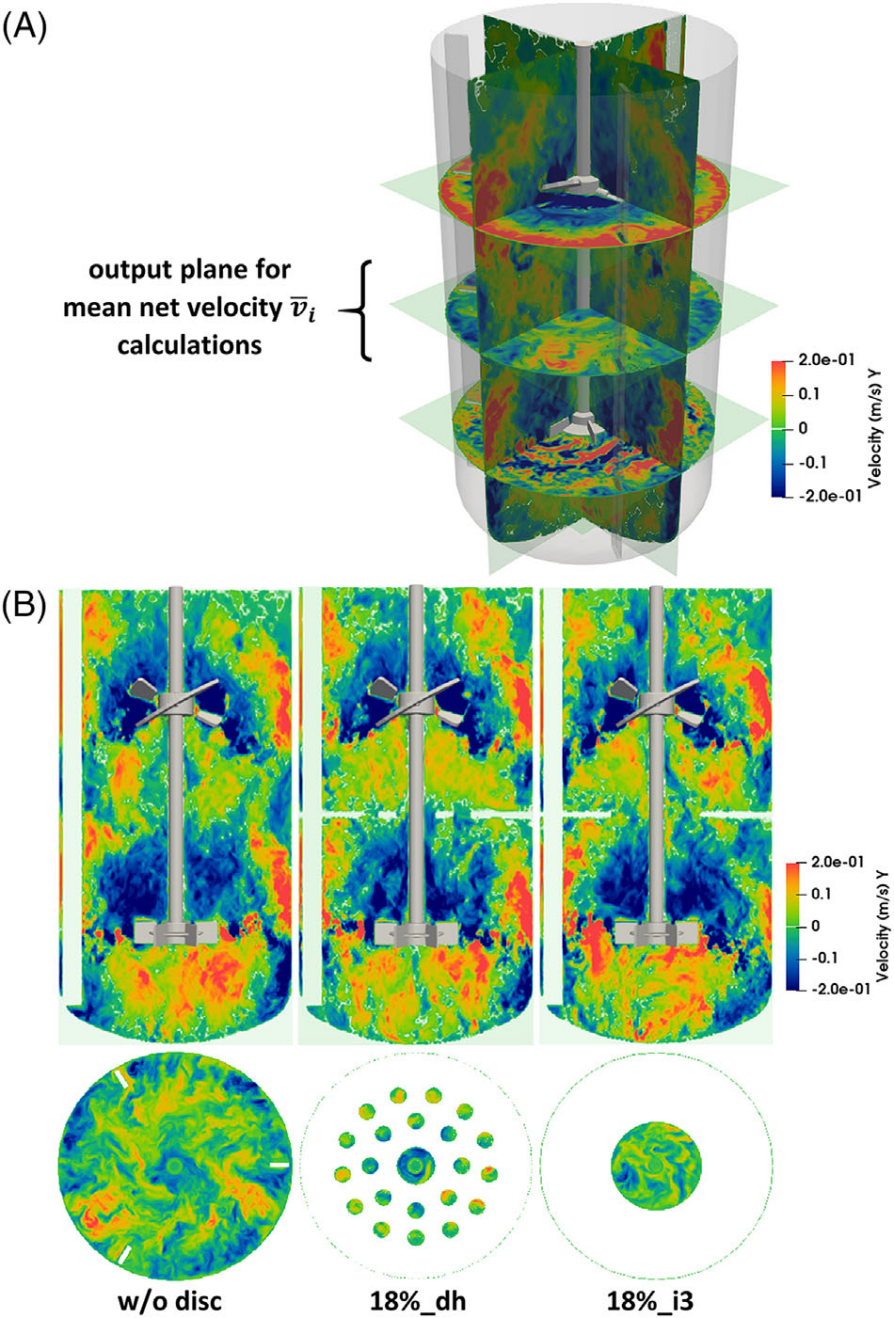
(A) 3D view of the small‐scale bioreactor with simulated vertical fluid velocities on 5 output planes. (B) Contour plots of vertical fluid velocities derived from (M‐Star) CFD simulations for the non‐compartmented reactor and the setups with compartment discs 18%_dh and 18%_i3

**FIGURE 5:**
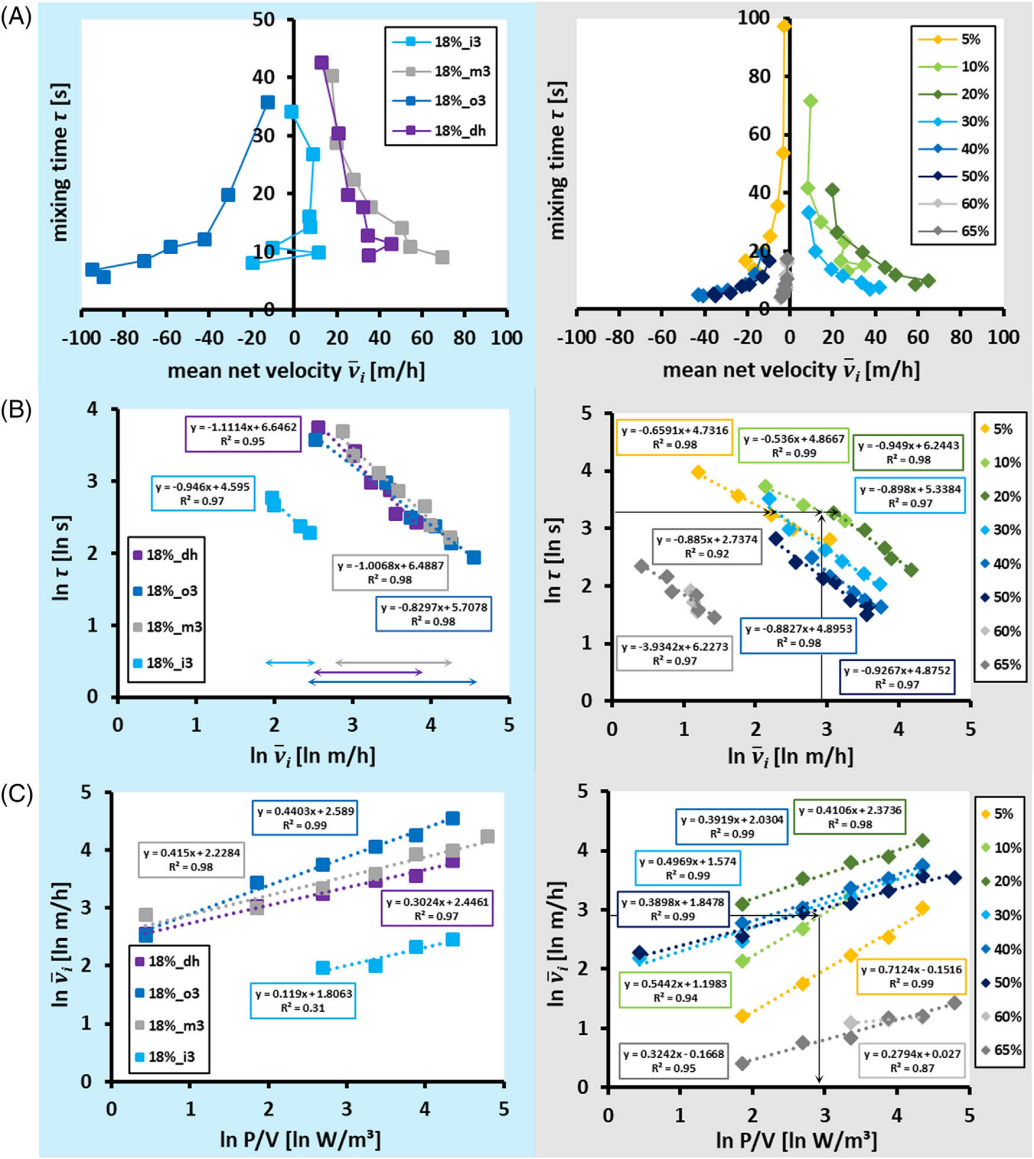
(A) Mixing times (*τ*) versus mean net velocities (${\bar{v}}_{i}$) for compartment discs differing either in the exchange area (*A_ex_*) provided between compartments (in %, grey side) or in the disc layout at a fixed *A_ex_* (blue side). ${\bar{v}}_{i}$ was calculated based on vertical fluid velocities derived from CFD simulations. (B/C) *ln‐*transformed ${\bar{v}}_{i}$ versus (B) *ln*‐transformed *τ* and (C) *ln*‐transformed volumetric power inputs (P/V). Data were fitted via linear regression

As shown earlier, both *A_ex_* and the location of *A_ex_* on the discs influenced the mixing behavior in the scale‐down reactor system. While the targeted adjustment of the mixing time via *A_ex_* could be established in Equation (6), a quantifiable factor to access the disc layout for the manipulation of mixing times remains to be identified. To this end, the vertical fluid velocities derived from the CFD simulations were considered. Contour plots of the simulated fluid velocities are shown in Figure 4B for the noncompartmented small‐scale bioreactor and for the compartment setups with discs 18%_dh and 18%_i3. Notably, the CFD results exhibited comparable velocity patterns for all the simulated setups. Consequently, to avoid CFD simulations for each new disc design, the velocities of the non‐compartmented small‐scale bioreactor were consulted, and the impact of the discs was included in the calculation. The velocity distribution of the non‐compartmented bioreactor was provided for the identical position of the cross‐sectional area where the discs were mounted (Figure 4A). Therefore, mean net velocities (${\bar{v}}_{i}$) were calculated for each disc and power input, as described in ‘Materials and Methods’. The results were plotted versus the mixing time to assess the strength and direction of the dominant flow through the respective discs (Figure 5A). Notably, negative rates code for a dominant downward flow while positive mean velocities encode predominant flows from the bottom to the top. The impact of the disc geometry may be best visible by analyzing the results of the 18%‐disc series, as depicted in Figure 5A. Only disc 18%_o3 showed a dominating downward flow, indicating that this flow direction dominates close to the bioreactor wall. In contrast, discs 18%_dh and 18%_m3 created upward flows, whereas disc 18%_i3 did not show a clear flow direction. Hence, the use of ${\bar{v}}_{i}$ is deemed to be a promising additional impact factor for selecting appropriate discs. It should be noted that large negative ${\bar{v}}_{i}$ values should shorten the mixing time in the LC.

Further steps included the *ln*‐transformation of the mean net velocities and plotting these versus similarly transformed *τ* and P/V. Again, well‐defined linear correlations were observed between *τ* and ${\bar{v}}_{i}$ and between ${\bar{v}}_{i}$ and P/V in double logarithmic plots. Interestingly, this was true for both the experimental series (Figure 5B and 5C). Notably, the majority of the linear models revealed *R* ^2^ ≥ 0.97. The identified linear correlations between *τ* and ${\bar{v}}_{i}$ or ${\bar{v}}_{i}$ and P/V are given by the following equations:

(7a) $$\ln\tau =\alpha^{*}\;\;\cdot \ln{\bar{v}}_{i}+\ln k^{*}$$

(7b) $$\ln{\bar{v}}_{i}=\alpha^{**}\;\;\cdot \ln\left(\frac{P}{V}\right)+\ln k^{**}$$

Through the double *ln*‐transformation, the algebraic sign of the velocities and the dominant flow direction was no longer accessible. However, in doing so, additional evaluation criteria were generated for the disc design. As indicated by the colored arrows for the 18%‐discs in Figure 5B, the disc designs differed in the velocity ranges that were available for a valid linear correlation with the mixing time, as stated in Equation (7a).

For example, for disc 18%_i3, a linear correlation between *τ* and ${\bar{v}}_{i}$ with a high *R* ^2^ was only observed for a narrowed selection of the conditions tested. Moreover, the conditions included in the correlation (P/V: 14.8–78.0 W/m^3^) with 7.2–11.6 m/h covered only a small range of velocities (see Figure 5B and C). In contrast, for the disc 18%_o3, a valid linear correlation was observed for the P/V range of 1.5–78.0 W/m^3^, excluding only the highest power input condition for which the mixing time already approached a constant value (Figure 2C). Furthermore, the included power input conditions created a comparably broad spectrum of mean net velocities between −12.5 and −94.9 m/h. Regarding future applications, these ranges could serve as a criterion to rank possible disc designs at a fixed *A_ex_*. In this example, it might be beneficial to favor the design of 18%_o3 over that of 18%_i3 to achieve higher correlations between τ and ${\bar{v}}_{i}$.

In summary, the findings of the 18% series, as shown in Figures. 5B and C allow the following conclusion: given the large‐scale *τ* and P/V, the *A_ex_* can be easily derived using Equation (6). The particularities of the disc design may be further deduced by using the impact factor ${\bar{v}}_{i}$ to arrive at a set of suitable disc designs. Therefore, a clearly contoured operational range for statistically sound application of the scale‐up setting has been provided in this study.

**FIGURE 6:**
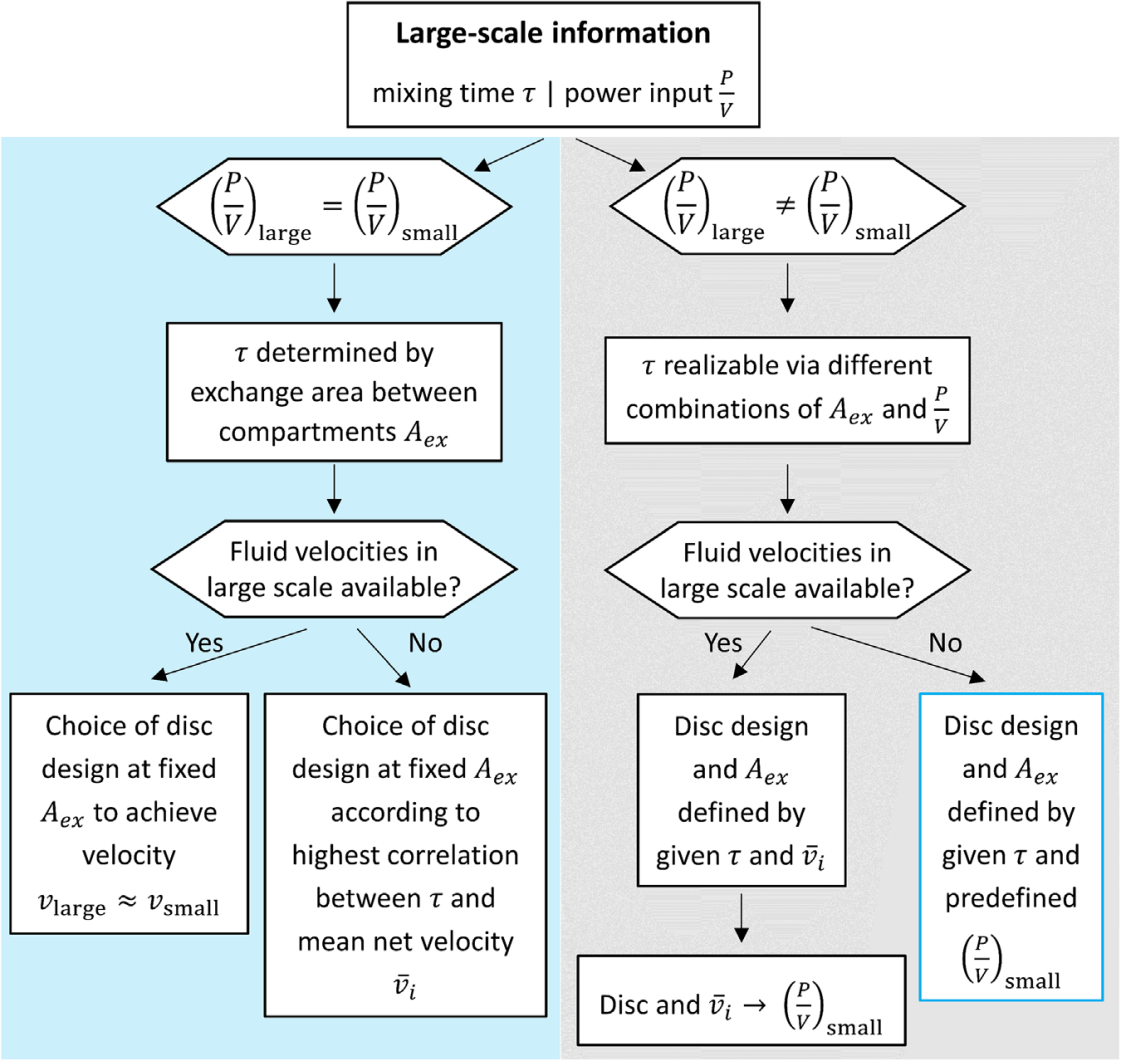
Decision tree for the selection of appropriate compartment disc designs using large‐scale mixing times as constraint and further considering mixing power input (P/V) and mean net velocity (${\bar{v}}_{i}$) as scale‐down factors

The mean net velocity ${\bar{v}}_{i}$ serves as an additional scale‐down factor, together with the mixing time and mixing power input. Consequently, different options were created to mimic large‐scale conditions in the SMCB. The alternatives were divided using the decision tree shown in Figure 6.

As mentioned before, *τ* derived from large‐scale measurements were taken as constraints and represented the starting point of any new system design. One possible method would be to use the power input (P/V) derived from a large‐scale process as the second constraint (Figure 6, blue side). This may be particularly true for large‐scale bioprocesses with low to moderate mechanical power input, as is the case in biopharmaceutical production using mammalian cell cultures. To realize the desired combination of *τ* and P/V, the required *A_ex_* can be calculated using Equation (6). In this scenario, all further modifications would then be realized at a fixed *A_ex_*, as exemplified in Figure 5, for a small selection of discs with a fixed *A_ex_* of 18% (Figure 5A–C, blue side). Depending on the availability of information on large‐scale velocities, the locations of cut‐outs on the disc could be distributed in a way that either achieves similar mean net velocities between the compartments on large and small scales (“Yes”‐path, Figure 6, blue) or achieves generally high correlations between *τ* and ${\bar{v}}_{i}$ (“No”‐path, Figure 6, blue).

Another method would be to choose the small‐scale power input separately from the large‐scale power input (Figure 6, grey side). In keeping P/V flexible, the same *τ*_large_ could be realized with different values of *A_ex_* (Figure 5B, grey). In this scenario, if large‐scale velocities are available, ${\bar{v}}_{i}$ can replace P/V as the second constraint. Thus, instead of applying ${\bar{v}}_{i}$ for smaller adjustments when finalizing the disc design, as described for P/V_large_ = P/V_small_, the disc design was chosen to create a target combination of *τ* and ${\bar{v}}_{i}$. An example is illustrated by the arrows in the grey Figure 5B. In this example, a disc with *A_ex_* = 10% was installed in the scale‐down reactor system to realize the depicted combination of *τ* and ${\bar{v}}_{i}$. The P/V set‐point required to create the previously discussed combination of *τ* and ${\bar{v}}_{i}$ with the given disc can then be derived from the correlation between ${\bar{v}}_{i}$ and P/V, as illustrated by the arrows in the grey Figure 5C. If it is desired to deviate from P/V_large_, even though no velocity data are available, the procedure starting with Equation (6) can be applied using a different P/V_target_ value. In addition, the aforementioned velocities were experimentally investigated using 4D‐Particle Tracking Velocimetry (4D‐PTV), as demonstrated by Kuschel et al. [27]. More detailed results on gaining insights into qualitative flow structures and coherent compartments are currently being evaluated and will be published soon.

### 3.4 Mixing times in the biphasic system

**FIGURE 7:**
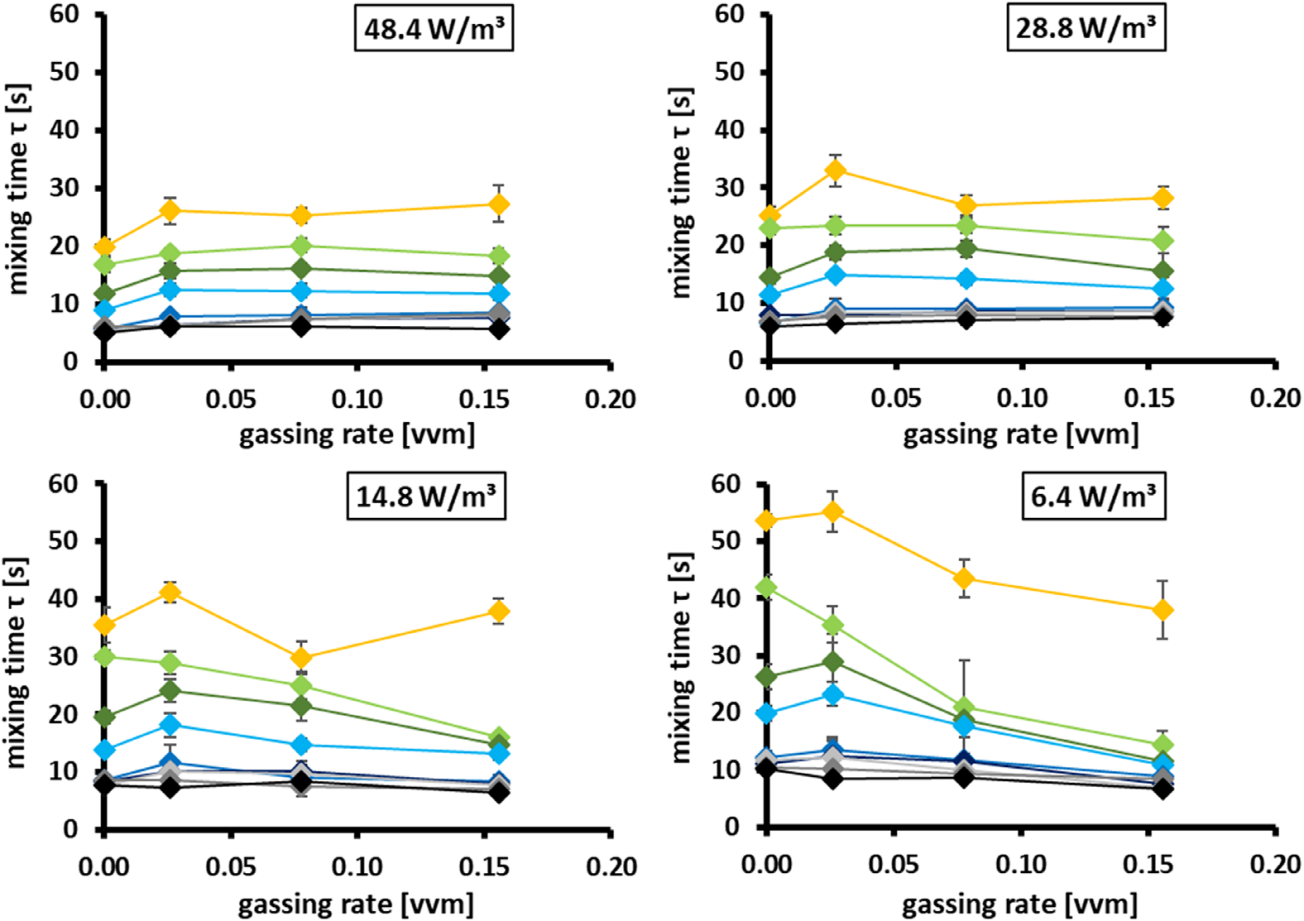
Gassed mixing times (*τ*) at 4 different power inputs for the noncompartmented bioreactor and for setups with different compartment discs providing exchange areas between 5 and 65% of the total cross‐sectional reactor area. Error bars indicate the standard deviation (*n* = 5)

To evaluate the extent to which the aforementioned design considerations are valid during cultivation in a gassed environment, mixing times were also determined in the biphasic system (Figure 7). Gassed mixing experiments were conducted at four different power inputs and gassing rates between 0.03 and 0.16 vvm to cover a typical gassing range during a CHO fed‐batch cultivation. Notably, for large‐scale, smaller gassing rates can be expected. However, higher gassing rates are necessary to ensure sufficient oxygen supply at the lab scale [28, 29].

At the two higher mixing power inputs, a slight increase in the mixing times was observed at the smallest gassing rate compared to the nongassed system. In addition, the mixing times remained at a comparable level for all gassing rates tested. Consequently, for scale‐down at higher power inputs, the discussed design correlations should remain valid during cultivation.

At lower mixing power inputs, enhanced gassing led to decreased mixing times and less pronounced differences between the discs 30–10% discs. However, the gassing rates of > 0.15 vvm at 6.4 W/m^3^ can be expected to represent the extreme case rather than a typical scale‐down scenario [23]. Nevertheless, even in this scenario, the 5% disc showed a significant increase in the mixing times. Hence, the scale‐down of comparable extreme power input and gassing rate combinations should be realizable with the system. The only drawback might be that the provided “one fits all”‐solution is not fully applicable for such cases, and more process‐individual disc designs might be necessary.

In the biphasic mixing time experiments, a small amount of gas bubbles was trapped below the disc, but no build‐up of the gas cushion could be observed. To a certain extent, an altered distribution of gas bubbles is desired to include zones of less sufficient oxygen transfer in the scale‐down model. Thus, it may be possible to recreate the effects of intermittent hypoxia, as described by Gao et al., at the production scale [30].

## 4 CONCLUDING REMARKS

Studies on the SMCB setting have shown that large‐scale mixing times can be easily installed by choosing an appropriate exchange area in the separating disc mounted between the upper and lower compartments. Hence, transferring large‐scale mixing times and integral mixing power inputs into the SMCB is possible for a wide range of disc designs tested. Further specification, which design is best suited to a particular scale‐down problem, may be easily gained by considering ${\bar{v}}_{i}$ as an additional impact factor. Therefore, the peculiarities of disc design for a given exchange area can be deduced. The individual findings were combined in a converging decision tree that enables the selection of the best feasible design by taking the large‐scale mixing time as a key criterion, which may be further specified with knowledge of volumetric power input and mean net velocities to further shortlist a set of disc design settings to a precisely contoured operational field of high statistical relevance. Notably, gassing tests showed that the basics of the SMCB design are not biased due to aeration.

Though the compartment configuration was kept constant in this study, follow‐up research will extend the proposed scale‐down approach by the impact of varying numbers and sizes of compartments. Considering residence time distributions and sizes of concentration zones, the SMCB might hold additional potential to further approximate large‐scale conditions.

The implementation of the SMCB approach would help advance novel applications in biopharmaceutical process analysis. To date, common scale‐down approaches have been hampered by the intrinsic bias of artificial shear stress imposed on cells circulating through the pumps of external loops. As the SMCB does not require related pumping, true readouts of cellular responses are envisaged in future scale‐down experiments. These should allow a non‐biased view of the cells to target the most sensitive mechanisms, rendering cells more robust for large‐scale applications.

## CONFLICTS OF INTEREST

The authors have declared no conflicts of interest.

## Supporting information

Table S1. Correlation factors *k* and *α* from empirical correlations between mixing time τ and volumetric power input P/V for compartment discs differing in the provided exchange area *A_ex_*

## Data Availability

The data that support the findings of this study are available from the corresponding author upon reasonable request.
