# Supplementary material for: Scaling‐down biopharmaceutical production processes via a single multi‐compartment bioreactor (SMCB)
Source: Eng Life Sci. 2022 Mar 14;23(1):e2100161. doi: 10.1002/elsc.202100161 (PMC9815078; doi:10.1002/elsc.202100161)
Supplement: Supplementary file 1 — Table S1. Correlation factors k and α from empirical correlations between mixing time τ and volumetric power input P/V for compartment discs differing in the provided exchange area Aex [file ELSC-23-e2100161-s001.pdf]

Table S1. Correlation factors *k* and *α* from empirical correlations between mixing time τ and volumetric power input P/V for compartment discs differing in the provided exchange area A_{ex}

A_{ex} [%]	α	k
5	-0.46	120.2
10	-0.40	86.2
20	-0.37	50.1
30	-0.37	38.8
40	-0.34	22.1
50	-0.30	19.4
60	-0.32	19.2
65	-0.32	20.0
